# Contribution of DA Signaling to Appetitive Odor Perception in a *Drosophila* Model

**DOI:** 10.1038/s41598-018-24334-x

**Published:** 2018-04-13

**Authors:** Yuhan Pu, Melissa Megan Masserant Palombo, Ping Shen

**Affiliations:** Department of Cellular Biology and Biomedical and Health Sciences Institute, University of Georgia, 500 D. W. Brooks Drive, Athens, GA 30602 USA

## Abstract

Understanding cognitive processes that translate chemically diverse olfactory stimuli to specific appetitive drives remains challenging. We have shown that food-related odors arouse impulsive-like feeding of food media that are palatable and readily accessible in well-nourished *Drosophila* larvae. Here we provide evidence that two assemblies of four dopamine (DA) neurons, one per brain hemisphere, contribute to perceptual processing of the qualitative and quantitative attributes of food scents. These DA neurons receive neural representations of chemically diverse food-related odors, and their combined neuronal activities become increasingly important as the chemical complexity of an appetizing odor stimulus increases. Furthermore, in each assembly of DA neurons, integrated odor signals are transformed to one-dimensional DA outputs that have no intrinsic reward values. Finally, a genetic analysis has revealed a D1-type DA receptor (Dop1R1)-gated mechanism in neuropeptide Y-like neurons that assigns appetitive significance to selected DA outputs. Our findings suggest that fly larvae provide a useful platform for elucidation of molecular and circuit mechanisms underlying cognitive processing of olfactory and possibly other sensory cues.

## Introduction

Olfaction is an ancient sense that is vital to the survival of animals across evolution. For example, to successfully search for distant or hidden energy sources in an ecological niche, a forager must be able to selectively recognize diverse food-related volatiles among others and evaluate their potential reward values. In addition, olfaction is also known to be functionally interconnected to other senses such as taste, and it may significantly impact cognitive functions that facilitate food detection and competition^1–4^. Therefore, elucidation of conserved neural substrates and mechanisms underlying cognitive processing of food odor stimuli in behaving animals is likely to provide valuable insights into the inner workings of the brain and its evolution.

An extensive body of knowledge is now available about the sensory processes within the initial two steps of the olfactory circuit. These studies have revealed a remarkable set of anatomical and functional similarities at multiple levels, including odor detection by olfactory receptor neurons, wiring schemes for relaying sensory inputs to second-order projection neurons as well as the presence of analogous spatial maps encoding processed odor information in the higher-order processing center (e.g., the lateral horn in flies and the cortical amygdala in mice that controls innate olfactory behaviors)^5,6^. In contrast, how such olfactory information is represented in deeper brain regions and translated to motivated behaviors remains poorly understood.

We recently developed a genetically tractable *Drosophila* larva model for investigation of perceptual processing of food-related odor stimuli^7^. Fly larvae have an advanced yet numerically simpler nervous system. Under well-nourished conditions, they display an aroused appetitive state in anticipation of sugar food following a brief presentation of a banana-like scent or balsamic vinegar vapor. This odor-induced attention to food reward involves an assembly of four third-order DA neurons that project their dendrites exclusively to the lateral horn in each brain hemisphere. By taking genetic, pharmacological and laser-based microsurgical approaches, here we show that in well-nourished larvae, a full cognitive capacity for attributing sugar reward to discrete olfactory cues requires the combined activities of clustered DA neurons. We provide evidence that these DA neurons define an odor perception module that has three key functions: serving as a convergence site for spatially segregated olfactory inputs from projection neurons, decoding and integrating signals representing chemically diverse odor stimuli of various strengths and transforming the processed information to one-dimensional DA outputs. Finally, we have also identified a higher cognitive process, gated by a D1-type DA receptor (Dop1R1) activity in neuropeptide F (NPF) neurons, for assigning appetitive significance to selected DA signals. Therefore, *Drosophila* larvae may provide a useful model for study the neurobiology and evolution of cognitive processing of appetitive sensory cues.

## Results

### Attribution of anticipated food reward to discrete odor stimuli

**Figure 1:**
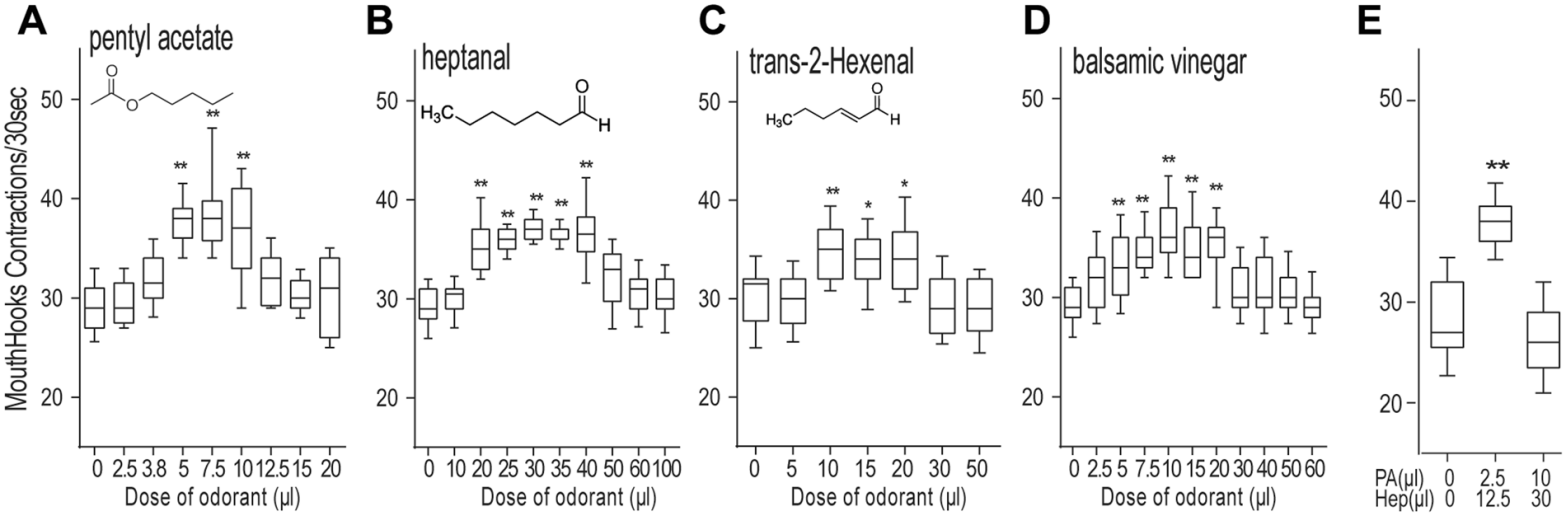
Attribution of anticipated food rewards to discrete odor stimuli by fed larvae. (**A**–**D**) Third-instar fed larvae (74 h after egg laying, AEL) were exposed to an odor stimulus for 5 minutes in a sealed chamber, fumigated with defined concentrations of monomolecular odorants PA, Hep, T2H or natural odorants balsamic vinegar, prior to the feeding test in 10% glucose liquid media. The odor effects on larval feeding rate were quantified by counting mouth hook contractions of each larva over a 30 s period. The rate of mouth hook contraction is positively correlated to the amount of dyed food ingested^7^. n ≥ 15. (**E**) Two binary odor mixtures were used instead: PA plus Hep and PA plus T2H. n ≥ 17. All behavioral quantifications for this and other figures were performed under blind conditions. Unless indicated otherwise, all the statistical analyses were performed using One-way ANOVA followed by a Dunnett’s test. *P < 0.05; **P < 0.001.

To investigate how fly larvae might recognize and respond to a variety of appetitive olfactory stimuli, we decided to quantify larval appetitive responses to three different monomolecular odorants, pentyl acetate (PA), heptanal (Hep) or trans-2-hexenal (T2H), that are known to be chemotactically attractive to *Drosophila* larvae^8^. Fly larvae use their external mouth hooks to scoop liquid food into the oral cavity, and the appetite of individual larvae can be reliably quantified by measuring changes in the rate of mouth hook contraction under blind testing conditions^7,9^. For example, a 20% increase in mouth hook contraction rate, induced by an appetitive banana-like scent, is equivalent to a 50–100% increase in dyed food ingestion^7^. By examining larval appetitive responses to increasing doses of each odorant, we have generated three dose-response curves (Fig. 1A–C). In each case, while the effective dose ranges of the three odorants differed significantly, their appetizing effects invariably followed an inverted-U function. In addition, balsamic vinegar vapor, a chemically complex odor mixture, also exhibited inverted-U effects (Fig. 1D). We also tested the appetitive responses of fly larvae to a number of binary mixtures. For example, when presented simultaneously, two separately ineffective doses of odors may become appetitive, while two separately appetitive doses may become ineffective (Fig. 1E). In combination, these results suggest that odor stimuli exert additive effects that may positively or negatively impact larval appetitive response. They also reveal that fed larvae have an innate cognitive ability to selectively extract salient features from a small fraction of odor stimuli that vary in chemical identity and strength.

### Convergence of food odor inputs onto the assembly of DA neurons

**Figure 2:**
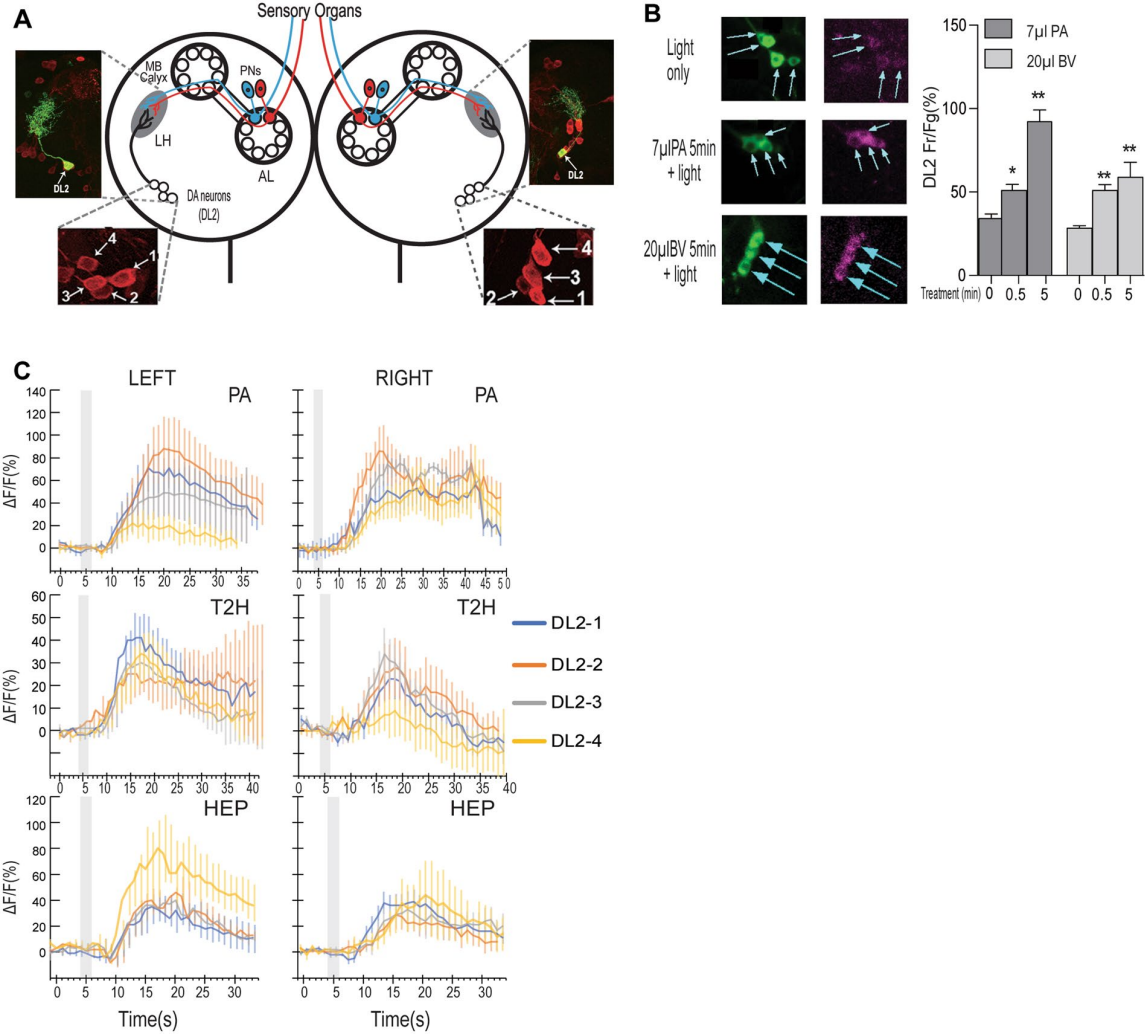
Convergence of food odor inputs onto the assembly of DA neurons. (**A**) Schematic drawing of the larval olfactory circuit shows two clusters of four DA neurons (DL2-1 to 4) in the left and right brain hemisphere^7^. The DL2 neurons project dendrites to the lateral horn (LH) in each brain lobe, where they form synaptic connections with the projection neurons. Upper insets show that the axons and dendrites of individual DL2 neurons, labeled with mCD8::GFP, are exclusively located in the LH region. AL: antenna lobe; MB: mushroom bodies; PNs: projection neurons. DL: dorsolateral. (**B**) CaMPARI-based fluorescence imaging of freely behaving larvae revealed the dynamic responses of four DL2 neurons to an effective dose of PA or balsamic vinegar (BV) during 5-minute stimulation. For each time point, at least 29 DA neurons were imaged. Data are presented as ± SEM. *P < 0.05; **P < 0.001 (**C**) GcaMP6 imaging revealed that in fed larvae, DL2 neurons from the left and right brain hemisphere showed differential excitatory responses to each of the three monomolecular odorants. The shaded box indicates the duration of odor stimulation. In each imaging assay, 5–6 neurons were examined for each cell type.

To understand how the brain mechanism for perceptual processing of the qualitative and quantitative attributes of food scents, we turned our attention to two clusters of DA neurons (named DL2-1 to 4) that are labeled by *TH-Gal4* (Fig. 2A)^7^. These DA neurons, which are essential for larval appetitive response to PA, project their dendrites exclusively to the lateral horn, and likely form synaptic connections with an unknown percentage of the 21 projection neurons that are unilaterally line wired to 21 olfactory receptor neurons^7^. The olfactory information, which may encode representations of tens or hundreds of individual volatile compounds, has been reported to be spatially segregated at the level of projection neurons^10,11^. To determine how the DL2 neurons receive a diverse array of olfactory inputs, we first examined the dynamics of the excitatory responses of the DL2 neurons to stimulation by PA or balsamic vinegar vapor in freely behaving fed larvae, using a fluorescent Ca^2+^ sensor named CaMPARI (Calcium Modulated Photoactivatable Ratiometric Integrator)^12^. Fed larvae were presented with an odor stimulus under the same conditions as described for feeding behavioral assays. After various odor treatments, the larvae were irradiated with 405 nm light for 3 seconds. This light irradiation irreversibly turns Ca^2+^-bound CaMPARI protein from green to red fluorescence, thereby capturing the excitatory state of the DL2 neurons in freely behaving larvae at the defined time point. The quantitative imaging analyses revealed that when stimulated by an effective dose of PA (7 µl) or balsamic vinegar (20 µl), the clustered DA neurons showed a gradual increase in intracellular Ca^2+^ level over a 5-min test period, as evidenced by increased ratios of red and green fluorescence signals in the DL2 neurons (Fig. 2B). These results provide *in vivo* evidence that all four DL2 neurons display simultaneous excitatory responses to a given food-related monomolecular odorant or odor mixture.

We also asked whether the four DL2 neurons within the left or right cluster respond to different odors in a uniform or differential manner. Filleted larval tissues expressing an *in vivo* Ca^2+^ indicator in projection neurons labeled by *GH146-Gal4* or *TH-Gal4* were imaged^13^. Under our imaging conditions, we observed the excitatory response of projection neurons to PA in fed larvae, as expected (Figure S1). We then examined how the clustered DL2 neurons respond to three distinct monomolecular odor stimuli, PA, Hep or T2H. We found that the excitatory responses to distinct odorants by individual DL2 neurons within each cluster are not always uniform (Fig. 2C). For example, the DL2 neurons in the left cluster showed more varied responses to PA or HEP, while those in the right cluster showed more similar responses. Together, these findings also raised a possibility that combined activities of the DL2 neurons may encode neural representations of different odor stimuli.

### Combined activities of four DA neurons in perceptual processing of odor stimuli

**Figure 3:**
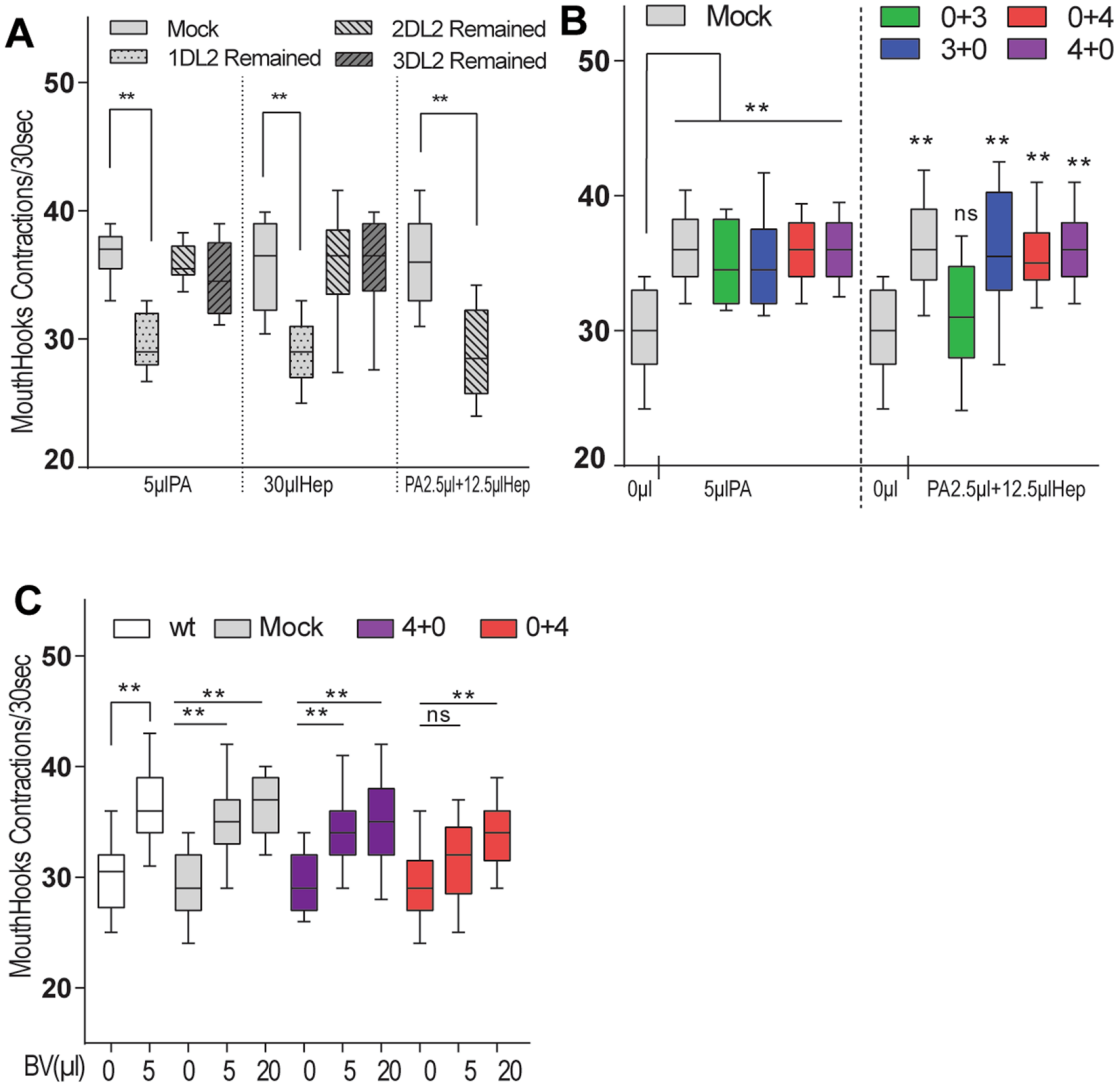
Combined activities of four DA neurons in decoding and integrating of odor stimuli. Targeted lesioning of individual DL2 neurons was performed in second-instar TH-GAL4/UAS-nlsGFP larvae by focused laser beams. After one day of recovery, freely behaving third-instar (74 h AEL) larvae were tested for the odor effects. The mock group was handled in the same way as that for experimental groups except for the laser treatment. (**A**) Three groups of experimental larvae, each having one, two or three DA neurons remained in the left or right cluster, were tested for the feeding response to an appetitive dose of PA or Hep or a binary mixture of PA and Hep (n ≥ 10). (**B**) Feeding responses to PA or a binary mixture by experimental larvae with three or four DA neurons in the right or left cluster. At least three DA neurons within the left cluster are required for the appetitive effect of the binary mixture (n ≥ 10). (**C**) BV induction of larval feeding response to 10% glucose media showed that a complete set of four DA neurons in the left cluster was sufficient to induce increased feeding, while four DA neurons in the right cluster showed weaker effects in the presence of lower effective doses of BV (n ≥ 12).

We also examined to what extent the two assemblies of DL2 neurons may work in coordination for perceptual processing of olfactory outputs from the sensory module. First, we functionally analyzed how many DL2 neurons are minimally required to mediate the appetitive response of fed larvae to monomolecular odorants and binary odor mixtures. By systematically lesioning these neurons through laser-mediated microsurgery, we found that the presence of two functional DA neurons within the left or right cluster was sufficient to mediate larval appetitive response to an effective dose of PA or Hep. However, when the monomolecular odorant is replaced with a binary mixture consisting 2.5 µl PA and 12.5 µl Hep, the minimal number of functional DA neurons required was increased to more than two (Fig. 3A). Further, we examined how four DL2 neurons within each cluster may function together when appetitive odor mixtures were used. For example, we found that the larval appetitive response to a binary mixture required at least three DL2 neurons in the left cluster or four DL2 neurons in the right (Fig. 3B). The larval appetitive response to balsamic vinegar vapor was also attenuated in the absence of the DL2 neurons in the left cluster (Fig. 3C). Importantly, this reduced appetitive response can be improved by increasing the appetitive dose of balsamic vinegar from 5 to 20 µl. Together, these results suggest that while the processing activity of each assembly of DL2 neurons is largely autonomously, the simultaneous actions of the left and right clusters appear to cause an additive effect that is particularly significant when appetitive odor mixtures are at relatively lower doses. Given that as few as four DL2 neurons are sufficient to mediate the appetizing effect of balsamic vinegar vapor, these results raise the possibility that fed larvae may perceive few chemical identities of balsamic vinegar. Consistent with this notion, the sparse glomerular responses to complex natural odors have been observed in the mouse olfactory bulb^14^.

### Appetizing doses of odor stimuli depend on the endogenous DA level

**Figure 4:**
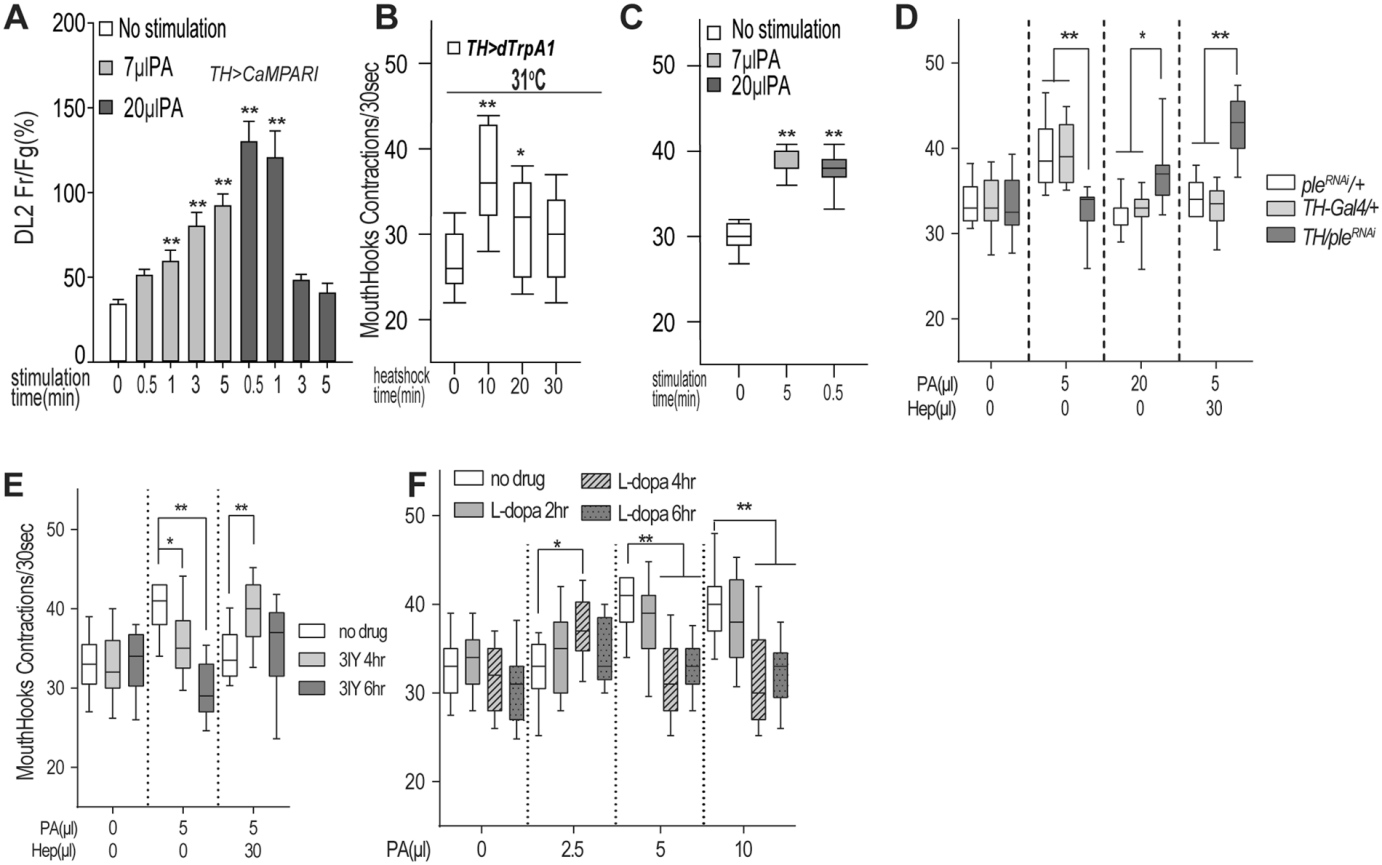
Appetitive odor stimuli trigger DA release from DL2 neurons within an optimum range. (**A**) In freely behaving larvae, DL2 neurons display distinct dynamic responses to an appetitive dose (7 µl) or non-appetitive dose (20 µl) of PA, as revealed by CaMPARI-based fluorescence imaging. Data are presented as ± SEM. n ≥ 7; (**B**) Genetic activation of fed larvae expressing the *dTrpA1* transgene at 31 °C for 10 minutes led to increased feeding response in the absence of odor stimulation (n ≥ 19). Also see Figure S1. (**C**) Fed larvae display appetitive responses to 30-sec stimulation by 20 µl PA, which is ineffective when stimulation time is 5 min (see Fig. 1A). n ≥ 11; (**D**) Expression of *TH RNAi* in *TH-*GAL4 neurons led to an increase in the minimal effective dose of odorants required for appetitive arousal (n > 12). (**E**) Wild type larvae pre-fed with 3IY-containing food for 4 hours also required a higher minimal effective dose of PA or a binary mixture for appetitive arousal (n > 11). (**F**) Wild type larvae pre-fed with L-dopa-containing food for 4 hours showed a reduction in the minimal effective dose of PA required for appetitive arousal (n > 9). **P < 0.001.

Since the appetizing effects of food-related odors invariably followed an inverted U function, we decided to assess how DL2 neurons contribute to differential responses to appetitive and non-appetitive odor stimuli in fed larvae. We found that in contrast to their gradually increased excitation over the 5-min stimulation by 7 µl PA, DL2 neurons stimulated by 20 µl PA showed a rapid surge of intracellular Ca^2+^ within the first minute, followed by a gradual reduction of the Ca^2+^ increase as the stimulation is extended (Fig. 4A). Therefore, the failure of 20 µl PA to arouse appetite may be accounted for by two opposing explanations. One possibility is that a 5-minute exposure to 20 µl PA may cause a stronger stimulation of DL2 neurons, which results in the suppression of acutely required DA signaling to downstream targets. Alternatively, the stimulation by 20 µl PA may cause an excessive release of DA, which is functionally ineffective. To distinguish the two possibilities, we first genetically activated the DL2 neurons over different time periods using dTrpA1, a temperature-sensitive TRP family cation channel (Fig. 4B)^15^. We found that a brief, but not a prolonged, activation of these neurons triggered an increased larval feeding response in the absence of odor stimuli. This observation argues against the notion that the silencing of the DA neurons per se is responsible for the non-appetizing effect of the 5-min simulation by 20 µl PA. To provide further evidence that the non-appetizing effect of 20 µl PA is caused by an excessive release of DA, we performed three additional tests. First, we stimulated fed larvae with 20 µl PA for 30 seconds instead. Indeed, fed larvae showed a significant increase in their feeding response (Fig. 4C), supporting the notion that the total amount of DA released by a 5-minute stimulation with 20 µl PA could be much higher than that with 7 µl PA.

We also modulated the endogenous DA level in fed larvae using molecular genetics and pharmacological approaches. To reduce the DA level, we performed RNA interference (RNAi)-mediated knockdown of Tyrosine Hydroxylase (TH), a rate-limiting enzyme for DA synthesis^16^. Fed larvae (*TH*-GAL4/UAS-*TH*^*RNAi*^) expressing a double-stranded RNA of *TH* in DA neurons failed to show significant appetitive responses to normally effective doses of odorants (e.g., 5 µl PA; Fig. 4D). However, the same larvae exhibited appetitive responses, when presented with higher, normally ineffective doses such as 20 µl PA or a binary mixture of 10 µl PA and 30 µl Hep. In parallel, larvae pre-fed with food containing a TH inhibitor, 3IY showed similar behavioral changes. For example, after pre-feeding with 3IY for 4 hrs, the larvae required abnormally high doses of PA or odor mixtures to trigger the appetitive response (Fig. 4E). In contrast, when the baseline level of DA was increased by pre-feeding with L-dopa, a precursor of DA^17^, larvae displayed opposite behavioral phenotypes (Fig. 4F). For example, after feeding in L-dopa-containing food for 4 hours, larvae showed appetitive responses to PA at a dose lower than normally required (e.g., 2.5 µl PA) instead. Therefore, L-dopa can augment lower doses of odorants to arouse larval appetitive response, and the minimal strength of an odor stimulus required for the arousal is inversely correlated with the baseline level of DA in fed larvae. Together, these findings strongly suggest that an excessively high level of DA, evoked by a strong odor stimulus, is not appetizing.

### Odor-induced DA signals acquire motivational salience through a Dop1R1 gating mechanism

**Figure 5:**
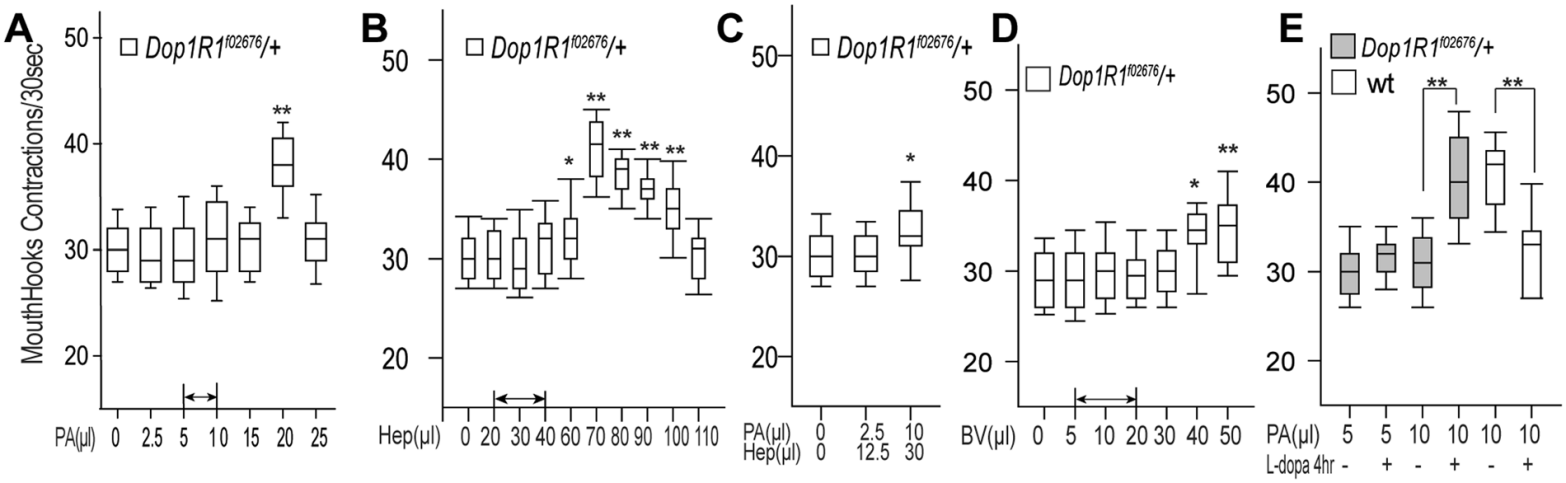
A Dop1R1 gating mechanism selective assigns motivational significance to a narrow spectrum of odor-induced DA signals. (**A**–**D**) Fed larvae heterozygous for *Dop1R1*^*f02676*^, a loss-of-function mutation in *Dop1R1*, showed a right shift in the inverted-U dose response when treated with PA, Hep, binary mix or balsamic vinegar vapor. The range of normally effective doses is indicated by arrows (n ≥ 11). (**E**) Feeding heterozygous DopR^f02676^ larvae with L-dopa for 4 hr restored the appetitive response to a normally effective dose of PA (n ≥ 13). *P < 0.05; **P < 0.001.

The genetic and pharmacological manipulations of DA signaling suggest that odor stimulation of various DL2 neurons causes increased DA releases into a common pool. We wonder whether an excessively large pool of odor-induced DA can become effective in fed larvae with a reduced level of DA receptor activity. *Dop1R1* was shown to be essential for the appetitive arousal of fed larvae^7^. To test this hypothesis, we used heterozygous *Dop1R1* fed larvae that have a 50% reduction in the level of *Dop1R1* transcripts^18^. In response to stimulation by various doses of monomolecular or mixed odorants, *Dop1R1*/ + larvae displayed a right shift in the dose-response curve (Fig. 5A–D). In addition, pre-feeding of L-dopa restored their appetitive responses to a normally effective dose of PA (Fig. 5E). These findings provide evidence that the DA/Dop1R1 signaling pathway defines a perception module that functionally links the olfactory and motivational process. It also suggests that DA outputs from the DL2 neurons have no intrinsic appetitive values, and a Dop1R1-gated mechanism attributes appetitive significance to selected DA signals evoked by appetitive odor stimuli.

### Functional mapping of the gating activity of Dop1R1

**Figure 6:**
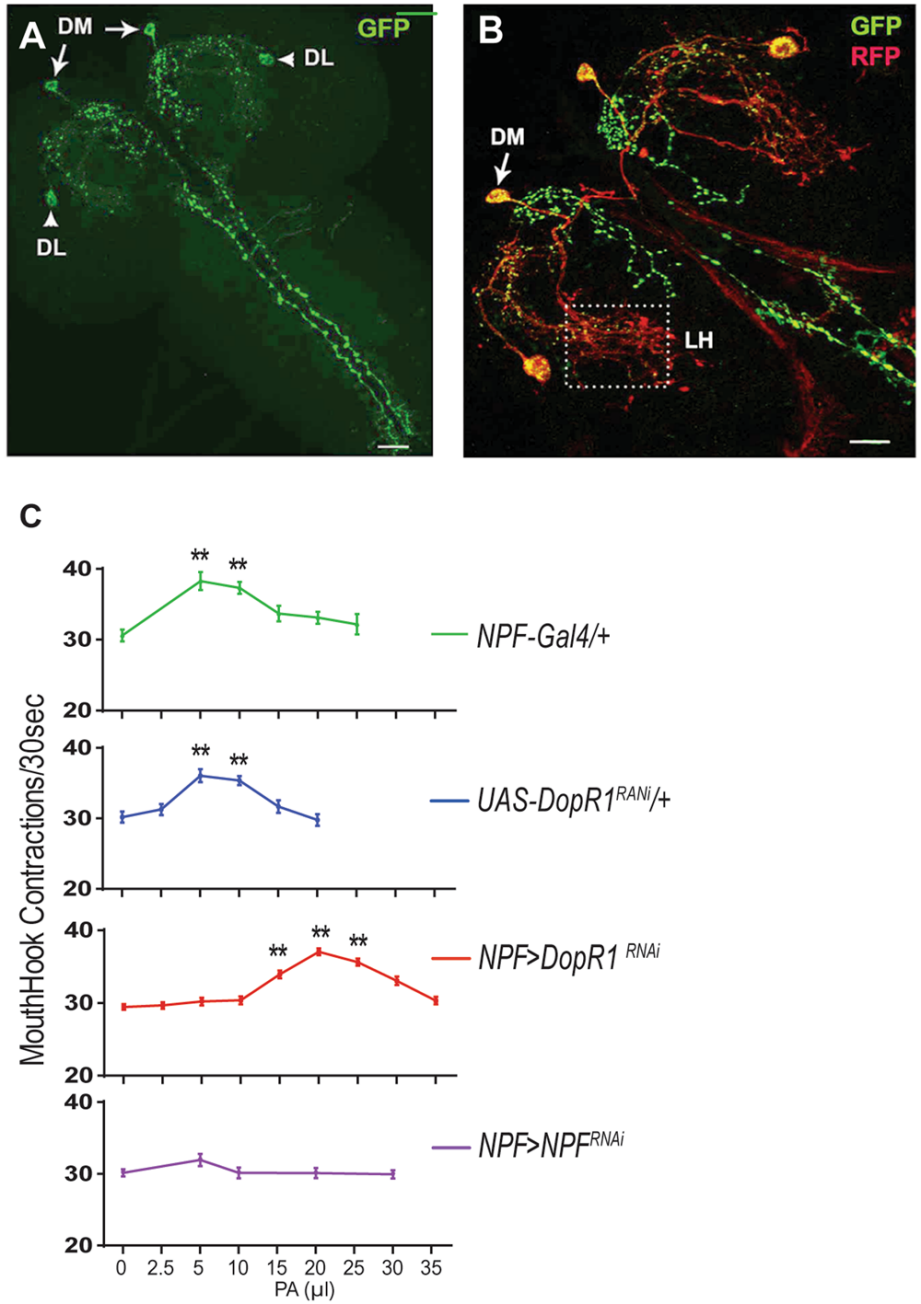
Mapping of the gating activity of Dop1R1 to NPF neurons. (**A**) An imaging of two pairs of neurons in the larval brain that are selectively labeled by *npf-*GAL4. DM: the dorsomedial pair; DL: the dorsolateral pair. (**B**) Expression of sytGFP in the axons (green) and denmark in the dendrite (red) of the *npf-*GAL4 neurons. There is an extensive presence of NPF neuronal dendrites in the lateral horn (LH). (**C**) The dose-response curves of *Dop1R1-*deficient, *npf-*deficient and control larvae (also see Figure S3 for other controls). n > 15; **P < 0.001.

It has been shown that NPF neurons are central to a reward system in *Drosophila*^19,20^. Therefore, the odor-evoked DA signals from DL2 neurons could potentially act upon the NPF-mediated reward circuit. A *npf-*GAL4 driver predominantly labels six NPF neurons in the larval central nervous system (CNS; Fig. 6A ^21,22^). We also observed an extensive presence of the dendrites of NPF neurons around the lateral horns Fig. 6B. To examine whether the gating activity of Dop1R1 resides in NPF neurons, we knocked down Dop1R1 activity in *npf-*GAL4/*Dop1R1*^*RNAi*^ fed larvae. Similar to *Dop1R1*/ + fed larvae, these *Dop1R1-*deficient larvae displayed a right shift of the dose-response curve (Fig. 6C, also see Figure S3). In addition, fed larvae expressing UAS-*npf*^*RNAi*^ under the direction of *npf-*GAL4 showed no significant appetitive response to odorants (e.g., PA and Hep) at any of the doses tested. Together, our findings suggest that odor-evoked DA signals arouse impulsive-like feeding in fed larvae by directly activating Dop1R1-gated NPF signaling.

## Discussion

We have performed a detailed functional analysis of the roles of two assemblies of third-order dopaminergic olfactory neurons and the activity of a DA/Dop1R1 signaling pathway in perceptual processing of appetitive odor stimuli in *Drosophila* larvae.

**Figure 7:**
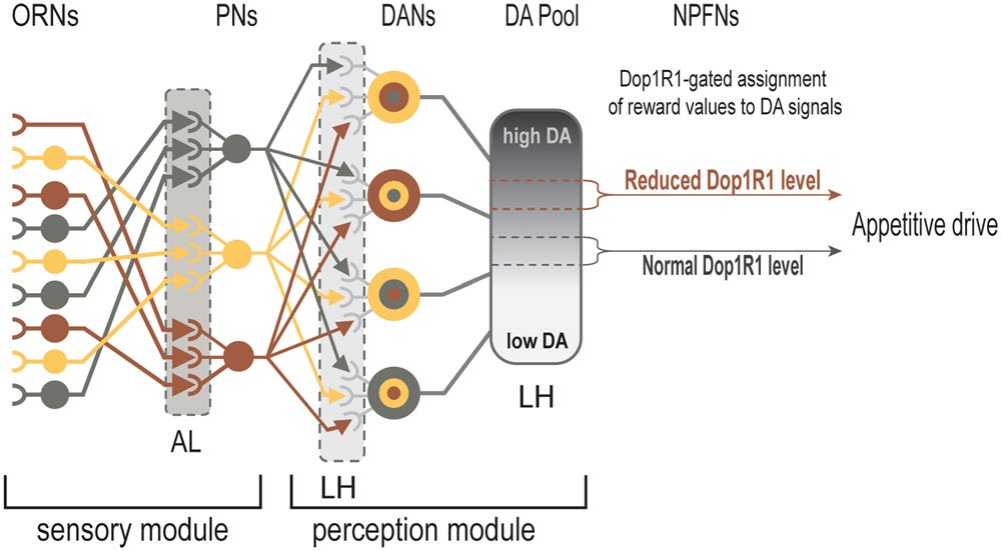
Schematic presentation of a neural circuit for sensation and perception of food-related odor stimuli. A the first part of the model depicts a previously characterized sensory module involving olfactory and projection neurons^7,33,34^. We posit that the four DA neurons in each cluster function in largely autonomous manner to integrate converging olfactory representations of various odorants, and a Dop1R1 activity-dependent gating mechanism in NPF neurons selectively assigns appetitive salience to a narrow spectrum of odor-induced DA signals. ORNs: olfactory receptor neurons; PNs: projection neurons; DANs: DA neurons; NPFNs: NPF neurons; AL: antenna lobes; LH: lateral horns.

Our findings have revealed that larval perception of appetitive food odors relies on four major functions of a DA/Dop1R1-mediated neural mechanism that likely resides in the NPF neurons (Fig. 7). First, in each brain hemisphere, an assembly of four DA neurons displays excitatory responses to a diverse array of individual monomolecular odorants or natural food scents in a differential manner. These neurons appear to serve as two parallel integration sites for a large number of spatially segregated outputs from second-order olfactory neurons^10^. It has also been reported that individual neurons in the olfactory cortex of the mouse receive diverse inputs from multiple mitral cells that represent a broad array of glomeruli^23^. We propose that the wiring organizations within the second-order olfactory processing centers of flies may also be similar to those in rodents.

Second, another major function involves the combinatorial activity of the clustered DA neurons in perceptual processing of converging olfactory inputs. By systematically lesioning individual DA neurons, we have shown that increasing numbers of DL2 neurons are required for the appetizing effects of odor stimuli as the chemical complexity of appetitive odors becomes higher. We also found that the appetizing effect of an odor mixture at relative lower doses is more sensitively affected by the partial loss of the DL2 neurons (Fig. 3). Together, these findings support the notion that the combined activities of the DL2 neurons may encode neural representations of different odor stimuli. Furthermore, sparse glomerular responses to complex natural odors have been reported in the mouse olfactory bulb^14^. Since similar numbers of DA neurons are required for the neural representations of an appetitive binary stimulus and complex balsamic vinegar vapor, these observations also suggest that fed larvae may perceive few chemical identities of balsamic vinegar.

The third major function of the DA mechanism relates to the compression of integrated odor signals to one-dimensional DA outputs by the clustered DA neurons. Genetic and pharmacological manipulations of the endogenous DA level showed that the minimal strength of an odor stimulus required to arouse appetite is inversely correlated with the baseline level of DA in fed larvae. For example, effective dose of an odor stimulus required for appetitive arousal can be significantly lowered in larvae pre-fed with L-dopa, or increased in larvae with reduced *TH* activity. Our findings also suggest that the appetizing level of odor-evoked DA signals may also subject to regulation by genetic factors that influence DA synthesis and transmission.

Finally, the Dop1R1-mediated circuit mechanism has a major role in the assignment of appetitive significance to selected DA outputs from the DL2 neurons. We have provided evidence that odor-evoked DA levels, when too high or too low, are not effective in arousing larval appetitive response. Furthermore, DA-coded olfactory information has no intrinsic appetitive values. When the Dop1R1 activity is reduced (Figs 5 and 6), the dose-response curves of all tested odorants showed a right-shift. On the other hand, administration of L-dopa to heterozygous *Dop1R1*larvae significantly restored the appetitive response (Fig. 5). These results suggest that the activity level of Dop1R1 defines a gating mechanism, which may control NPF neuronal signaling, that predetermines which range of DA signals may acquire appetitive significance.

The inverted-U effects of DA on cognitive functions have been widely observed in humans and other animals^24,25^. Malfunctioning dopamine systems also underlie many psychiatric disorders such as schizophrenia^26,27^. In the prefrontal cortex of mammals, an optimum level of D1-type DA receptor activity is crucial for spatial working memory, while its signaling at levels that are too low or too high leads to impaired working memory^28–30^. Therefore, our findings raise the possibility that a homologous DA receptor-mediated tuning strategy may be used to mediate the inverted-U effects of DA in flies and mammals.

## Materials and Methods

### Fly Stocks and Larval Growth

All flies are in the *w*^1118^ background. Larvae were reared at 25 °C, and early third instars (~74 hr *a*fter *e*gg *l*aying, AEL) were fed before behavioral experiments as previously described^7^. The transgenic flies include *TH-*GAL4^16^, UAS-*dTrpA1*^31^, UAS-GCaMP3 (BL32116), UAS-GCaMP6(BL42749), UAS-*TH*^*RNAi*^ (BL25796), and *npf-*GAL4 were obtained from the Bloomington Stock Center. UAS-*Dop1R1*^*RNAi*^ (V107058) was obtained from the Vienna *Drosophila* RNAi Center. *DopR*^*f02676*^ flies were described previously^18^.

### Behavioral Experiments

Quantification of mouth hook contraction rate in liquid food was performed as previously described^19^. A published protocol for fly larvae odor stimulation was used with slight modifications^7^. Briefly, synchronized early third instars, fed on yeast paste, were stimulated for 5 minutes with specified doses of single or mixed odors including pentyl acetate (PA) (Sigma-Aldrich, 628-63-7), heptanal (Hep) (Sigma-Aldrich, 117-71-7), trans-2-Hexen-1-al (T2H) (Sigma-Aldrich, 6728-26-3), and the vapor of balsamic vinegar or ripen pineapple juice. After rinsing with water, larvae were tested for their feeding responses. The liquid medium for feeding assays contains agar paste (US Biological, A0940) and 10% glucose. UAS-*dTrpA1* was expressed by allowing larvae to feed in pre-warmed yeast paste in a 31 °C incubator for defined periods, followed by rinsing with 31 °C water prior to feeding assays.

### 3IY and L-dopa Feeding

3-iodo-L-tyrosine (3IY) (Sigma-Aldrich, 70-78-0) and L-DOPA-ring-d_3_ (L-dopa) (Sigma-Aldrich, 53587-29-4) were used. The protocols for administration of 3IY and L-dopa were described previously^7^. The concentrations of 3IY and L-dopa in yeast paste were 10 mg/ml and 0.5 mg/ml, respectively.

### Immunostaining

Tissue dissection and fixation and antibodies were described previously^7^.

### Targeted Laser Lesion

Protocols for calibration of 337 nm nitrogen laser unit and laser lesion experiments have been described^32^. Selection of the DA neurons for laser lesioning was based on their unique morphological features described previously^7^.

### Functional Imaging

Processing of intact tissues of the larval nervous system and GCaMP6-mediated imaging of DA neurons that project to the lateral horns was performed as previously described^7^. The conditions of larval feeding and odor treatment for CaMPARI imaging are identical to those for odor-aroused feeding behavioral assays. After odor stimulation, larvae were irradiated with PC light 405 nm LED array (200 mW/cm^2^, Loctite) for 5 s. The treated larval CNS was dissected and individually scanned using a Zeiss LSM 510 confocal microscope.

### Statistical analysis

Statistical analyses for behavioral, CaMPARI imaging assays were performed using One-way ANOVA followed by Dunnett’s or Sidak’s multiple comparisons test.

### Data availability statement

The datasets generated during and/or analysed during the current study are available from the corresponding author on reasonable request.

## Electronic supplementary material

Supplementary info
