# Supplementary material for: Contribution of DA Signaling to Appetitive Odor Perception in a Drosophila Model
Source: Sci Rep. 2018 Apr 13;8:5978. doi: 10.1038/s41598-018-24334-x (PMC5899149; doi:10.1038/s41598-018-24334-x)
Supplement: Supplementary file 1 — Supplementary info [file 41598_2018_24334_MOESM1_ESM.pdf]

Supplementary Information

Contribution of DA Signaling to Appetitive Odor Perception in a *Drosophila* Model

Yuhan Pu[1], Melissa Megan Masserant Palombo and Ping Shen*

[*1]Department of Cellular Biology and Biomedical and Health Sciences Institute, University of Georgia, 500 D. W. Brooks Drive, Athens, GA 30602, USA

* Correspondence should be addressed to P.S. (pshen@uga.edu)

Fig S1 The excitatory response of GCaMP3-labeled dendrites of multiple projection neurons in the glomeruli of the antennal lobe

It has been reported that olfactory representation is spatially segregated at the level of projection neurons [10,11]. Left panel: an example of an intact CNS tissue showing fluorescent GCaMP3-labeled dendrites of multiple projection neurons, marked by *GH146-Gal4*, in a subset of glomeruli of the antennal lobe; right panel: calcium imaging showed that only a small subset of the projection neurons were excited by brief PA stimulus, as indicated by the pseudocolor image of GCaMP3. Scale bar =20μm.

Fig S2 Control Larvae Display Normal Feeding Responses after Heat Shock Treatment.

Fed larvae from three control groups display normal feeding responses after heat shock at 31°C. n > 15.

Fig S3 The dose response curves of *Dop1R1*-defieicent and *npf*-deficient larvae and controls

(A) *Dop1R1*-deficient fed larvae in the presence of Hep. **(B)** The control larvae (UAS-*NPF*^{RNAi} alone) in the presence of PA. **(C, D)** *NPF*-deficient fed larvae in the presence of Hep or a mixture of PA and Hep. n>14; **P<0.001

Fig S1

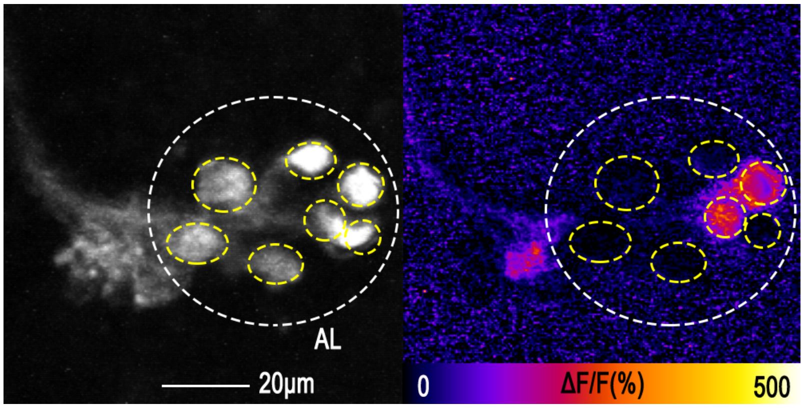

Fig S2

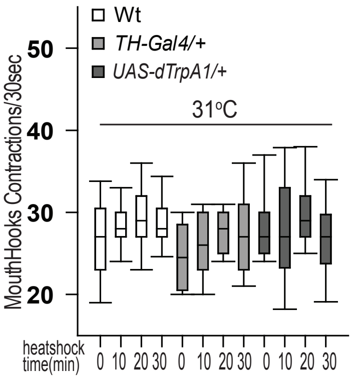

Wt
TH-Gal4/+
UAS-dTrpA1/+
31°C
MouthHooks Contractions/30sec
50
40
30
20
heatshock time(min)
0 10 20 30 0 10 20 30 0 10 20 30

Fig S3

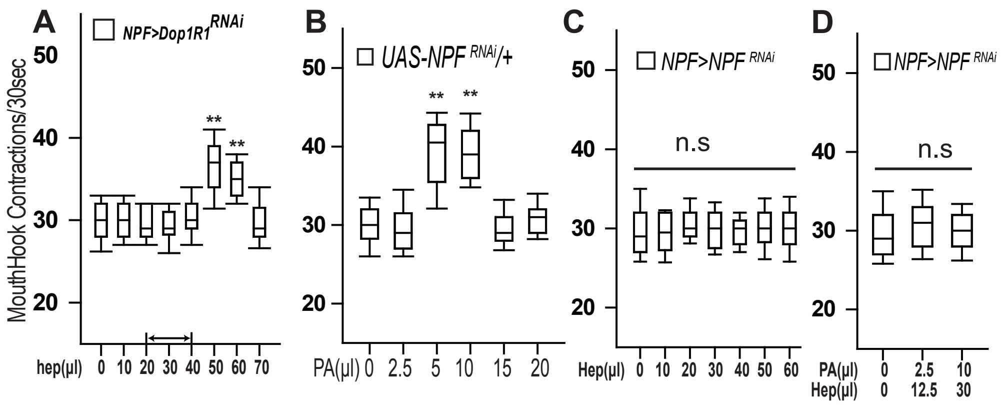
A
NPF>Dop1R1 RNAi
MouthHook Contractions/30sec
50
40
30
20
**
**
hep(µl) 0 10 20 30 40 50 60 70
B
UAS-NPF RNAi/+
50
40
30
20
**
**
PA(µl) 0 2.5 5 10 15 20
C
NPF>NPF RNAi
50
40
30
20
n.s
Hep(µl) 0 10 20 30 40 50 60
D
NPF>NPF RNAi
50
40
30
20
n.s
PA(µl) 0 2.5 10
Hep(µl) 0 12.5 30
